# Wearable 3-D Photoacoustic Tomography for Functional Brain Imaging in Behaving Rats

**DOI:** 10.1038/srep25470

**Published:** 2016-05-05

**Authors:** Jianbo Tang, Jason E. Coleman, Xianjin Dai, Huabei Jiang

**Affiliations:** 1 J. Crayton Pruitt Family Department of Biomedical Engineering, University of Florida, Gainesville, FL 32611, USA; 2 Child Health Research Institute, Department of Pediatrics, College of Medicine, University of Florida, Gainesville, FL 32610, USA; 3 School of Physical Electronics, University of Electronic Science and Technology of China, Chengdu, Sichuan, China

## Abstract

Understanding the relationship between brain function and behavior remains a major challenge in neuroscience. Photoacoustic tomography (PAT) is an emerging technique that allows for noninvasive *in vivo* brain imaging at micrometer-millisecond spatiotemporal resolution. In this article, a novel, miniaturized 3D wearable PAT (3D-wPAT) technique is described for brain imaging in behaving rats. 3D-wPAT has three layers of fully functional acoustic transducer arrays. Phantom imaging experiments revealed that the in-plane X-Y spatial resolutions were ~200 μm for each acoustic detection layer. The functional imaging capacity of 3D-wPAT was demonstrated by mapping the cerebral oxygen saturation via multi-wavelength irradiation in behaving hyperoxic rats. In addition, we demonstrated that 3D-wPAT could be used for monitoring sensory stimulus-evoked responses in behaving rats by measuring hemodynamic responses in the primary visual cortex during visual stimulation. Together, these results show the potential of 3D-wPAT for brain study in behaving rodents.

Imaging in behaving animals is becoming an important tool in behavioral neuroscience and preclinical brain disease therapy studies. It offers scientists the opportunity to correlate brain function with voluntary sensorimotor responses, which is not feasible in traditional anesthetized or head-fixed preparations^1^,^2^. *In-vivo* micro-electrode arrays are capable of recording neuronal action potentials at high speed in behaving rats^3^,^4^, but it is invasive and often restricted to limited number of recording electrodes^5^. Conversely, functional magnetic resonance imaging (fMRI)^6^, positron emission tomography (PET)^7^, and diffuse optical tomography (DOT)^8^ offer 3-D noninvasive recording of whole brain metabolic/hemodynamic responses in awake/behaving animals, but their spatial and/or temporal resolution is relatively low (millimeters for fMRI, PET and DOT; seconds for fMRI and PET). Optical microscopic imaging techniques, such as two-photon microscopy (TPM)^9^, can provide high spatial (micrometers) and temporal resolution (milliseconds), and have been used to examine brain function-behavior in behaving animals in proof-of-principle studies^10^,^11^. Although less invasive than micro-electrode methods, it still runs a risk of damaging the brain vasculature or neurons, thereby affecting its efficacy in chronic studies. In addition, the imaging region in TPM is generally limited to several square millimeters (1–4 mm^2^). Moreover, the high optical scattering in biological tissues restricts the imaging depth to <1 mm. Therefore, an imaging technique with high spatiotemporal resolution that can noninvasively image neural function across the brain, including substantial depth along the dorsoventral axis, in a behaving animal is in high demand.

Photoacoustic imaging is an emerging technique that has been widely used in biomedical applications^12^,^13^. In principle, absorption contrasts within the tissue are acoustically detected via the photoacoustic effect in which initial acoustic pressure arises when chromophores undergo a heat increase after absorbing the incident light energy^14^. By selecting the light wavelength, specific absorption agents can be identified due to their different absorption coefficients, e.g. deoxyhemoglobin (HbR) is more sensitive at 710 nm while oxyhemoglobin (HbO) is more sensitive at 840 nm^15^. Therefore, spectral photoacoustic technique is capable of functional imaging^16^. Acoustic transducer array-based photoacoustic tomography (PAT) is one implementation of photoacoustic technique that produces images by tomographically acquiring the whole ‘plane’ acoustic signal at one laser pulse^14^. It is particularly suitable for animal brain imaging since it can noninvasively image an animal’s brain at centimeter penetration with micrometer-millisecond spatiotemporal resolution^17^,^18^,^19^. However, the current array-based PAT systems are bulky, requiring subjects to be anesthetized or restrained, which can possibly affect the animal’s perception to stimulation, resulting in stress and suppression^17^,^20^,^21^. For example, there is evidence suggesting that anesthesia can affect neuronal excitability, cerebral hemodynamics, vascular reactivity, cerebral metabolism, and other baseline physiological metrics^22^,^23^,^24^.

In our previous work, a wearable photoacoustic tomography (wPAT) was developed for brain imaging in behaving rats^25^. However, the animal’s vision was impaired because its eyes were blocked beneath the probe, and the animal was confined to linear motion (forwards and backwards) due to the light delivery strategy used. Moreover, the previous wPAT was a 2D imaging technique since it had only one elevation layer of fully functional acoustic transducer array. Thus, two major obstacles must be overcome before wPAT can be fully realized in behaving animals. First, achieving 3D imaging is recognized as an important step for brain imaging because vascular regulation appears to initiate within the middle and deeper layers of the brain^26^. Second, the wPAT must allow an animal to explore its surroundings with free visual interaction.

In this paper, we present a wearable 3D photoacoustic tomography (3D-wPAT) technique that can noninvasively image a behaving rat’s brain with high spatiotemporal resolution. The 3D-wPAT had three elevation layers of fully functional acoustic transducer array that was comprised with 3 × 64 elements. The transducer array was arranged into a 2/3-ring-shaped casing that can be mounted on the rat’s head and allow the eyes to be unblocked. The light delivery interface and the passive weight support system gave the experimental animal a large mobility during study. The 3D-wPAT was capable of imaging object at high spatial resolution (~200 μm in-plane spatial resolution), and can acquire a 3D brain image within 167 ms (no averaging). In proof-of-principle experiments, we tested the 3D imaging ability of the 3D-wPAT via phantom and animal experiments and demonstrated its advantage in the application of functional 3D brain imaging in behaving rats via a hyperoxia experiment. In addition, to our knowledge, we show for the first time that this novel 3D-wPAT permits studies of the visual system in behaving animals by demonstrating that 3D-wPAT is capable of detecting visually evoked responses in the primary visual cortex (V1).

## Results

### 3D Wearable photoacoustic tomography system

**Figure 1:**
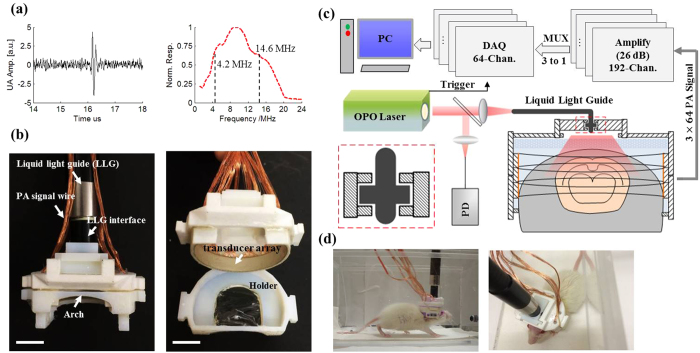
(**a**)Measured ultrasound signal (left) of one acoustic transducer element and its frequency response (right). (**b**) Photographs of the fabricated 3D-wPAT probe. Length of scalebar: 10 mm. (**c**) Schematic diagram shows the light illumination, light energy monitoring and data amplification and acquisition system. The insertion schematic shows the rotatable LLG interface design. PD: photodiode. (**d**) Photographs show the behaving ability of a rat while wearing 3D-wPAT.

The 3D-wPAT probe contains an array of 3 × 64 PVDF-based (Polyvinylidene fluoride) acoustic transducer elements (Fig. 1(b)). Measurement of the array’s property provided a central frequency of 9.6 ± 0.12 MHz (mean ± s.e.m., n = 192) with a bandwidth of 96.4 ± 2.8% (mean ± s.e.m., n = 192, −6 dB level) (Fig. 1(a) shows the ultrasound signal (left) and frequency response (right) of one representative transducer element). As shown in Fig. 1(b), the probe casing was designed to have an inner diameter of 36 mm, outer diameter of 43 mm, and an overall height of 20 mm. It was comprised of three assemblies, including a holder, a ‘cap’, and the liquid light guide interface (LLG interface). The holder was used to mount the probe to the animal head and was designed to have a head-shaped hole in the center that was covered with a transparent plastic membrane (Home-Use Plastic Wrap, Stretch-tite Inc., Sutton, MA); and two pairs of threaded holes, through which screws could be bi-laterally pressed against the bone near the ear. The ‘cap’ was used to house the acoustic transducer array (right, Fig. 1(b)). The casing was prototyped with 3-D printing using polyamide material (online 3D Printing Service, I.materialise.com), and had a total weight of ~20 g (excluding cablings).

Figure 1(c) presents the schematic diagram of light excitation, PA signal detection, and data amplification and acquisition. The pulsed laser light (repetition rate: 20 Hz, pulse duration: 10 ns, wavelength range: 680–2550 nm, model: Surelite OPO Plus, Continuum Inc., San Jose, CA, USA) was split into two beams by a beam splitter (ThorLabs Inc. Newton, NJ, USA). The reflected beam was recorded by a photodiode for light energy monitoring; the transmitted beam was coupled into the proximal end of a liquid light guide (LLG, core diameter = 8 mm, NA = 0.59, model# 77631, Newport Inc., Irvine, CA, USA), which had its distal end connected to the LLG interface. Signals were recorded via a custom built data amplification and acquisition system. This system contained 192 signal amplifying channels (26 dB) that were 3-to-1 multiplexed (MAX 4051, MAXIM Inc., San Jose, CA, USA) into 64 data acquisition channels (10 bit, 50 Ms/s, PCIAD 850, US Ultratek Inc., Concord, CA, USA) (see Wang *et al.*^21^ for detail). Each laser pulse triggered the DAQ module to acquire a 64-channel signal that was detected by one layer transducers of the 3 × 64-element array. Therefore, a 3D brain image could be acquired through three laser pulses. With a dedicated passive weight support system (Methods) and the rotatable design of the LLG interface (close-up insertion in Fig. 1(c)), the animal can move forwards, backwards and turn around fully during experiments, as shown in Fig. 1(d).

### Phantom evaluation of 3D-wPAT

**Figure 2:**
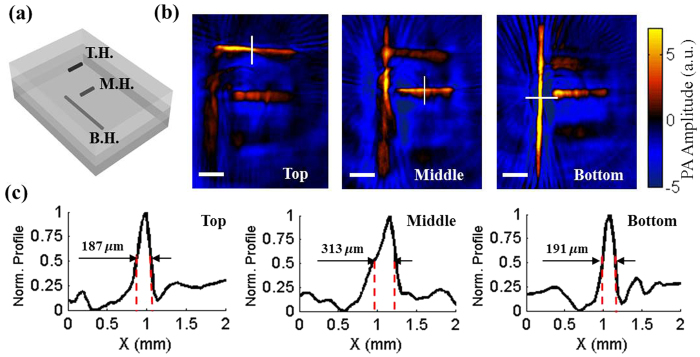
(**a**) Schematic diagram shows the phantom sample that was embedded with three hairs at three different depths. B.H.: bottom hair buried at depth of ~5 mm; M.H.: middle hair buried at depth of ~3 mm; T.H.: top hair buried at depth of ~1 mm. (**b**) wPAT images acquired by the acoustic transducer array’s top, middle, and bottom layers. Excitation light wavelength: 797 nm. Length of scale bar: 1 mm. (**c**) Profile and FWHM measured at the middle point of each hair shown in (**b**).

As an initial test, the 3D imaging ability of 3D-wPAT was evaluated with a phantom sample (background optical absorption coefficient: 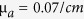; reduced scattering coefficient: 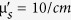). It was designed to have three hairs (diameter: ~80 μm) embedded in three depths (1 mm, 3 mm, and 5 mm, respectively), as shown in Fig. 2(a). The irradiation light wavelength was 797 nm and the light intensity was measured to be ~2.5 mJ/cm^2^ per pulse. Fig. 2(b) presents the wPAT images acquired by the acoustic transducer array’s top, middle, and bottom layers, respectively. The results showed that the top, middle, and bottom hairs were successfully reconstructed in their corresponding acoustic detection layer while blurring in other layers, demonstrating that the developed device is capable of 3D imaging. In addition, five transects’ (equal interval) value of full width at half maximum (FWHM) for each hair were measured from the corresponding layer’s wPAT image, resulting in values of 207 ± 7.8 μm, 293 ± 9.1 μm, and 228 ± 12.4 μm (mean ± s.e.m., n = 5), respectively. Fig. 2(c) shows the profiles along the white lines marked in Fig. 2(b) for each hair. The larger measured FWHM of the middle hair may be attributable to the fact that the middle hair was not placed at a depth precisely equal to the middle acoustic detection layer, which could have compromised the shape of its normalized profile curve relative to the other hairs. Nonetheless, these results indicate that the 3D-wPAT can achieve an in-plane X-Y spatial resolution of ~200 μm. Since the current 3D-wPAT had only three acoustic detection layers (each has a height of 2 mm), it was difficult to render a 3D stacked image to measure a precise Z-axis resolution. We estimated that its Z-axis resolution was ≥2 mm, which was primarily determined by the element height of the acoustic transducer array.

We further evaluated the ability of 3D-wPAT in detecting an object with changing concentration/optical absorption coefficient (i.e. varied absorption contrast to background). The bottom ultrasound detection layer of the transducer array was used to image the object since the lowest level of light fluence transferred to this layer, which represents the worst condition for 3D-wPAT imaging.

**Figure 3:**
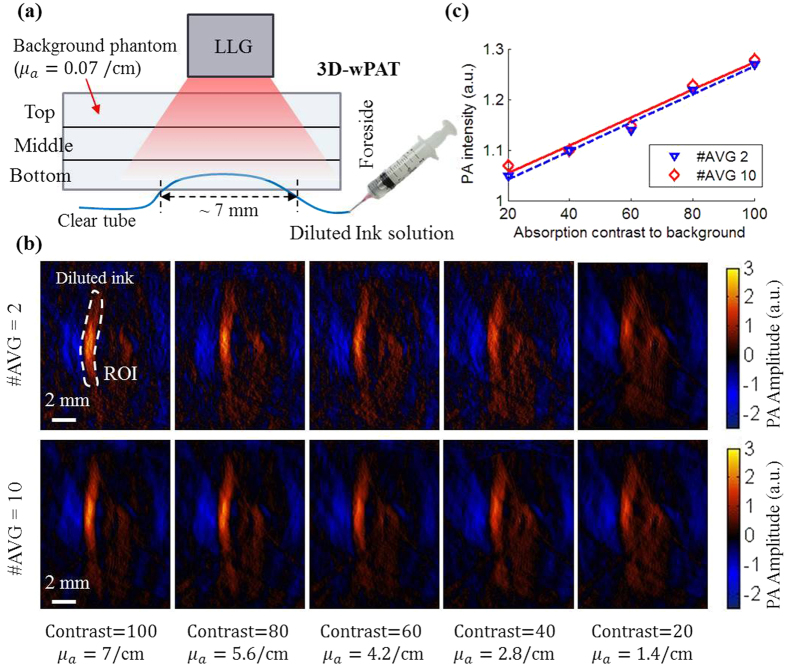
(**a**) Experimental setup. (**b**) wPAT image obtained by the bottom ultrasound detection layer. (**c**) Mean PA intensity of the ROI with respect to absorption contrast and averaging times. #AVG: times of averaging.

As shown in Fig. 3(a), a clear tube (inner diameter: 1 mm) was buried in a background phantom (optical absorption coefficient: μ_a_ = 0.07/cm; reduced scattering coefficient: 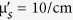). The tube was placed within the bottom ultrasound detection layer of the transducer array at a depth of 4–5 mm and the length of the tube within the imaging plane is around 7 mm. Diluted ink solutions (optical absorption coefficient: 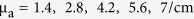, i.e. absorption contrast to background = 20, 40, 60, 80, and 100, respectively) were injected into the clear tube with a syringe. Figure 3(b) shows wPAT images obtained with twice averaged (top) and ten times averaged (bottom), respectively. White dashed circle shows the target region (ROI) which was in a good agreement with the actual ‘tube’ target in terms of its diameter, length and shape. The target was successfully identified in all cases. It was also noted that, by increasing the concentration/optical absorption coefficient of the ink solution, the target showed a higher image intensity. Figure 3(c) shows the calculated mean PA intensity of the ROI with respect to different solution concentration/absorption contrasts and averaging times. It can be found that the PA intensity increased along with the increase of ink concentration/absorption contrast, and it was linearly related to the target’s absorption contrast for both twice averaged and ten times averaged cases. This experiment shows that 3D-wPAT can successfully detect the object’s concentration change/optical absorption variation, suggesting it is able to detect changes in oxygen saturation/HbO concentration.

### Hyperoxia imaging in a behaving rat

We then evaluated the functional imaging ability of 3D-wPAT in a behaving rat undergoing hyperoxia. Since oxyhemoglobin (HbO) and deoxyhemoglobin (HbR) are the two dominate absorbing agents in blood, two wavelengths are enough to determine cerebral hemodynamic oxygen saturation (sO_2_). We selected wavelengths of 710 nm (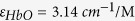, 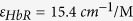) and 840 nm (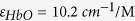, 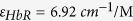) for light excitation^15^. The experiment was performed as follows: for every recording time point, 3 times-averaged (i.e. ~0.5 s to acquire a whole 3D brain image) 840 nm 3D-wPAT images were obtained immediately following 710 nm excitation, and vice versa. Therefore, the time interval between each sO_2_ map was around 1+5 = 6 s (acquisition of 3 times-averaged images at 840 nm and 710 nm took 1 s in total, and 5 s was the time required for laser wavelength tuning).

**Figure 4:**
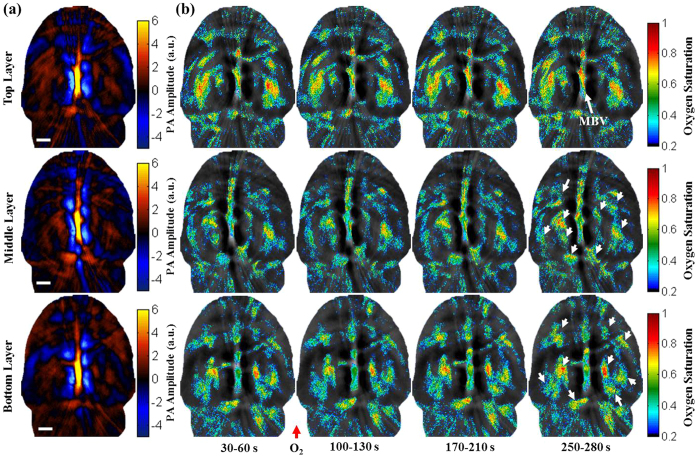
Functional imaging using multi-wavelength light excitation (710 nm and 840 nm). (**a**) Total cerebral hemoglobin wPAT images obtained from each layer in a behaving rat. (**b**) Calculated in-plane oxygen saturation map for each acoustic detection layer. Oxygen gas was applied to the experimental chamber at the 100th second. MBV: middle blood vessel. Length of scale bar: 1 mm.

The animal was placed in an experimental chamber (Fig. 1d) and pure oxygen was pumped into the chamber 100 s after the start of the experiment. The experiment duration was 300 s, and a total of 100 3D-wPAT images (50 images for each wavelength) were acquired. Figure 4(a) presents the total cerebral hemoglobin wPAT images, and Fig. 4(b) shows the calculated sO_2_ maps for each acoustic detection layer. It should be noted that the sO_2_ ratio increased throughout the brain vasculature after being supplied with oxygen. Differences were also noted between the three layers. In the top ultrasound detection layer, there was a trend for increasing sO_2_ that was detected only in the middle blood vessel (MBV). The mean sO_2_ ratio in MBV was greater at the end of experiment relative to baseline (from 0.48 to 0.71). In comparison, recordings across the brain in the middle and bottom layers showed a trend of increasing sO_2_. As shown in Fig. 4(b), the regions marked with white arrows underwent increases in oxygen saturation, and they were spread across the whole deep and middle brain. Qualitatively, it is also apparent that sO_2_ maps in the middle and bottom layers increased from light-blue to green-yellow, suggesting that oxygen saturation increased across deep brain regions. These results are consistent with the idea that hemodynamic changes initiate in medial and ventral brain regions^26^, reinforcing the importance and significance of deep-penetration 3D brain imaging.

### Visual stimulation imaging in behaving rats

**Figure 5:**
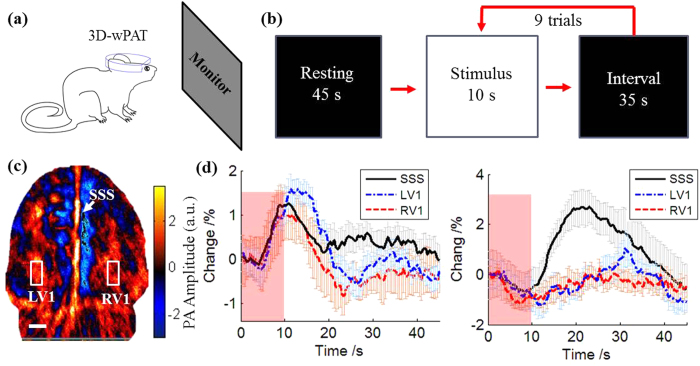
Visual stimulation experiment. (**a**) Experiment setup. (**b**) Flash stimulation paradigm. (**c**) The wPAT image from top layer (cortex layer). Three ROIs were marked on the wPAT image. RV1: right primary visual cortex (V1); LV1: left primary visual cortex (V1); SSS: superior sagittal sinus. (**d**) Cerebral blood vessel (CBV) response averaged across the 9 trials (superimposed with standard error of the mean, n = 9) for all ROIs. Right, CBV response under 710 nm light excitation (HbR); Left, CBV response under 840 nm light excitation (HbO). Length of scale bar: 1 mm. The red shading represents 10 s stimulus.

We next assessed the ability of 3D-wPAT in imaging visually-evoked hemodynamic changes in V1. While the subject’s head direction was fixed to face the monitor, the animal was able to move forwards and backwards a short distance (~5 cm). An LCD monitor (36 cm × 30 cm) was placed 20 cm in front of the animal as shown in Fig. 5(a). The monitor was controlled by PsychoPy^27^ to generate a flash visual stimulus. Figure 5(b) shows the stimulation paradigm that, in the first 45 s, the monitor was set to black screen, and then followed by 9 trials of stimuli switching between white screen (10 s) and a black screen (35 s). The experiments were performed in a darkened room. The top ultrasound detection layer of the 3D-wPAT was selected for recording cortical hemodynamic response. The wPAT images were acquired with 3 times averaging, and the imaging rate was around 6 frames/second (accounting the time consumed by data acquisition and saving).

Figure 5(c) shows the reconstructed wPAT image of the cortex layer, from which the cerebral vasculatures were readily evident. Three ROIs, including the right primary visual cortex (RV1, 0.8 × 1.6 *mm^2^*), left primary visual cortex (LV1, 0.8 × 1.6 *mm^2^*) and the superior sagittal sinus (SSS, 0.6 × 7 *mm^2^*), were chosen for analysis of the temporal evolution of hemodynamic responses. The data in Fig. 5(d) were obtained by averaging across the 9 trials (superimposed with standard error of the mean (s.e.m.)) for 710 nm light excitation (HbR) and 840 nm light excitation (HbO), respectively. There was a notalbe HbR change in all three ROIs following the flash onset (Fig. 5(d), right panel), all of which showed a notalbe increase above baseline within 10 s that decayed back to baseline within 20 s. In contrast, there was a modest change in HbO in all 3 ROIs following the flash onset (Fig. 5(d), left panel), but only the LV1 and RV1 ROIs displayed a sustained decrease below baseline within 10 s that recovered back to baseline within 20 s, which mirrored the HbR. These results match those that would be predicted by the fact that local V1 neurons are excited by the flash stimulus, and, in general, exhibit increase firing rates, thereby consuming more oxygen, which would result in the increase of HbR and decrease of HbO. The SSS is a major vein receiving blood flow from the cerebral blood vessels (CBV). Therefore, the parallel increase in HbR and decrease in HbO is reasonable as there should be an overall increase in oxygen concomitant with visual stimulation. However, the HbO response measured in SSS (Fig. 5(d), right panel) displayed an increase immediately following the end of the flash stimulus, which was different than the other rat’s result (Sup Fig. 1b, right panel), showing the complexity and challenge of performing experiment in awake behaving animal. Overall, the LV1 and RV1 signals were consistently phase-locked to the state of the visual flash stimulus, demonstrating high specificity in recording visually evoked activity. Notably, functional magnetic resonance imaging studies have also reported similar phenomenon in V1 and SSS regions in anesthetized rats^28^. These results were replicated in the other rat experiment (Sup Fig. 1).

## Discussion

We have developed a novel 3D-wPAT system that permits noninvasive brain imaging with hundred micrometer-scale spatial resolution and sub-second temporal resolution. The innovative implementation of the PVDF film for fabricating the acoustic transducer array paves the way for imaging in behaving subjects using photoacoustic tomography. The rotatable LLG interface and the passive weight support system allows for a large degree of mobility for animal’s wearing the device. Further, the 2/3-ring-shaped probe design circumvents the problems with earlier designs that obstructed the visual field of the subject. We demonstrated the ability of the new 3D-wPAT probe to monitor cerebral hemodynamics across the entire anterior-posterior extent of the cortex, as well as across the dorsal-ventral axis deep in the brain (~5 mm) in a behaving rat. We also demonstrated the utility of 3D-wPAT for studying 3D functional hemodynamic responses in the brain of a behaving animal via the hyperoxia experiments. Moreover, to our knowledge, this is the first demonstration of the potential for using photoacoustic imaging to monitor visually evoked responses in behaving rats.

The 3D-wPAT is currently limited to three ultrasound detection layers due to the limitations of wiring such a dense array in a small space coupled with the tradeoff between element sensitivity and the reduction in element size. One solution to overcome these limitations would be to use piezo ceramics (PZT) material which shows much higher electric-mechanical coupling coefficient. However, PZT is brittle, expensive, and has higher acoustic impedance compared to PVDF, tissue and water. Another possible improvement would be to employ the flexible printed circuit board (Flex PCB) for backing and dense signal wiring^29^. Flex PCB allows easy transducer shaping and high density wiring by utilizing the multilayer technique. However, the connection from the transducer element to PCB pad would be challenging. The novel Z-axis/anisotropic conductive adhesive/film is a feasible solution for connecting the arrays’ electrode surface to the conductive tracks or pads on Flex PCB^30^. The X-Y plane spatial resolution could also be improved by increasing the number of elements in each layer, but also introduces the challenge of dense signal wiring. Additional improvements could be made to significantly increase the functional imaging rate. The manual switching of laser wavelength to detect the brain’s oxygen saturation change greatly reduced the imaging rate in the current hyperoxia experiment. To address this problem, two trigger-sequence-controlled lasers^16^ or fast wavelength switch lasers could be employed, which are now commercially available. Despite the miniaturized size and reduced weight of the probe, the rotatable design of the LLG interface, and the utilization of the passive weight support system, the moving distance and environment that the animal can explore were still limited. The primary restraint was introduced by the LLG/fiber bundle, which was heavy and difficult to bend. One solution to minimize the restraint could be to employ a diameter reduced -high efficiency liquid light guide coupled with an optical system that expands the beam to the animal head homogeneously.

Generally, rats weighing 50–120 g were used for noninvasive photoacoustic tomography^18^,^20^,^21^,^25^ since the thick and opaque skull of an adult rat (weight ~200 g) would significantly reduce the light energy transmitted through the skull, thus decreasing the signal-to-noise-ratio (SNR). If minimal invasive procedure (i.e. removal of the scalp and thinning the skull) is acceptable, 3D-wPAT can also be used for studies performed using adult rats. The visual stimulus-evoked hemodynamic responses detected by 3D-wPAT were relatively weak and required averaging across 9 trials. However, trial averaging of visually evoked signals is a common practice during electrophysiological and optical imaging experiments, partly account for noise, but also to account for variability in neural responses that can vary due to a wide number of reasons including the animal’s attentiveness to the stimulus. Nonetheless, improvements to the transducer element’s sensitivity and the addition of a more stable laser, signal amplification, and data acquisition system should improve the ability of this technique to capture relatively weak hemodynamic responses with less (or without) trial averaging. Limited by the working range of the LLG (600–800 nm, 75% transmittance), 840 nm wavelength light was selected to detect the HbO response in the visual test experiment. Although the change in HbO was successfully detected, the PA signal drop was weak during stimulation since the optical absorption coefficient of HbO is only about twice to that of HbR at 840 nm wavelength. A much longer wavelength working range LLG should be employed in future experiments so that 1,000 nm wavelength light (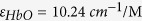, 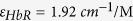) could be used to detect HbO response. As discussed above, two trigger-sequence-controlled lasers or a fast tuning multi-wavelength laser could be adopted to detect the oxygen saturation responses, which is more appropriate for functional brain imaging.

In conclusion, a novel 3D wearable photoacoustic tomography system has been developed and verified via phantom and behaving animal experiments. Notably, the 3D-wPAT could be readily adapted for use in imaging cerebral hemodynamics in behaving mice by designing an appropriate bottom mount holder suitable for a smaller head. In summary, in addition to being robust and cost-effective, the 3D-wPAT system developed here has the potential to serve as a powerful tool for the neuroscience community by providing noninvasive 3D brain images at high spatiotemporal resolution in behaving rats and mice.

## Methods

### 3D-wPAT design and passive weight support system

**Figure 6:**
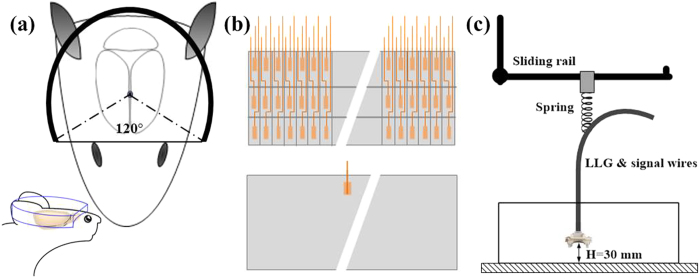
(**a**) Schematic diagram shows the design of 3D-wPAT which was a 2/3-ring in shape. (**b**) Schematic diagram shows the 3 × 64-element acoustic transducer array that was homemade with PVDF film. Top, divided positive face having 3 × 64 acoustic transducer elements; bottom, shared negative face. (**c**) Schematic diagram of the passive weight support system.

To achieve the goal of 3D brain imaging in behaving animals, a novel design of a miniaturized photoacoustic tomography combining a 3 × 64-element acoustic detection probe and a rotatable light delivery interface was proposed. As illustrated in Fig. 6(a), the acoustic transducer array was designed to cover a detection angle of 240° so that the animal’s vision would not be impaired while the brain was still within the imaging field. Furthermore, by designing an ‘arch’ on the foreside face of the casing, the forebrain deeper layers’ photoacoustic (PA) signal could be collected by the transducers since the probe can be moved down to sufficiently cover the foreside deep brain, as shown in Fig. 6(a) (left bottom) and Fig. 1(b). Polyvinylidene fluoride (PVDF, Measurement Specialties, Inc., Hampton, VA) film was selected to fabricate the acoustic transducer array since it is uniquely flexible, lightweight, and possesses a high coupling efficiency to tissue^31^,^32^,^33^, making it suitable for the 3D-wPAT ultrasonic detection. Moreover, the PVDF-based acoustic transducer array can be fabricated via an innovative design by producing transducer elements on a single piece of PVDF film. As illustrated in the top panel of Fig. 6(b), the positive face of a 110 μm thick PVDF film (76.8 × 6 mm^2^) was divided into 3 × 64 cells (element size, 1.2 × 2 mm^2^; etching width, ~100 μm). Then the 3 × 64-element acoustic transducer array was produced by simply sharing the negative face, as shown in the bottom panel of Fig. 6(b). With this design, the acoustic transducer array could be homemade at a very low cost and the property difference between each element was minimized. The acoustic transducer array was then glued into the ‘cap’ housing and 5-Minute Epoxy gel (Devcon Inc. Danvers, MA, USA) was used to isolate the acoustic array. In addition, a passive weight support system was designed to reduce the exertion upon the animal when wearing the device. As shown in Fig. 6(c), it contained a low elasticity spring (k = 10N/m), a slipping ring, and a sliding rail. All cablings were suspended by the spring and the 3D-wPAT was lifted to a height of ~30 mm. The equivalent weights exerted to the animal at the heights of 30 ± 20 mm were ±20 g, respectively (generally, the vertical movement of rat head was within ±10 mm).

### Animal preparation

Male Sprague Dawley rats (50–80 g, Harlan Labs, Indianapolis, IN) were used for this study. Two pairs of screws were used to mount the bottom holder of 3D-wPAT (Fig. 1(b)) on the rats’ head after the hair was removed (while the scalp and skull were still intact). The screws were bi-laterally pressed against the bone near the ear area (there was no surgery performed). In order to protect the skin, mounting tape (Scotch Inc., Los Angeles, CA) was placed between the screws and rat head. The head-shaped hole of the holder (Fig. 1(b)) was covered with a clear membrane (Home-Use Plastic Wrap, Stretch-tite Inc., Sutton, MA) so that the phantom solution could be solidified without leakage. The rat was allowed to acclimate to the device for one hour prior to the experiment.

One rat was used for the hyperoxia experiment. The animal was placed in the experiment chamber (Fig. 1(d)), in which it was able to move around during experiment. A plastic membrane (Home-Use Plastic Wrap, Stretch-tite Inc., Sutton, MA) was used to cover the experiment chamber to prevent possible oxygen leakage. After 100 s resting state, pure oxygen was continually aired into the experiment chamber for 200 s.

Two rats were used for the visual stimulation experiments. The rats were habituated to head-restraint condition (i.e., fixed facing direction, starting at 5 min and progressing up to 30 min). Restraint was positioned so that the animal faced the LCD monitor by fixing the LLG interface; it therefore could not turn around but it could move forwards and backwards approximately 5 cm during experiment. Each visual stimulation session lasted 450 s (1 session consisted of 45 s of a black screen followed by 9 trials of 10 s of white screen/35 s of black screen). Following one session at one wavelength, the rat was released for ~5 min and re-applied with 3D-wPAT for experiment at another wavelength.

The maximum laser intensity used for animal experiments under 710 nm and 840 nm were measured approximately 6 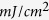 per pulse at the rat head surface (~1.5 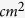). It was below the suggested maximum exposure of 20 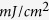 for a single pulse specified by the American National Standards Institute (ANSI)^34^. The 6–7 mm-high space between the probe and the rat head was filled with clear phantom solution (i.e., distilled water +1% agar power) which served as an impedance matching medium for PA signal. In addition, a 2 mm-thick scattering-only phantom layer (reduced scattering coefficient 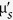 = 10/cm) was attached to the top of the clear phantom to achieve a more homogenous light distribution. The phantom solution (~55 °C) was solidified within 3–5 mins in the room temperature (~25 °C).

All procedures were approved by the University of Florida Animal Care and Use Committee and conducted in accordance with the National Institutes of Health Guide for the Care and Use of Experimental Animals.

### Data processing

Offline data processing was performed with MATLAB (Mathworks, Natick, MA). All A-line signals were bandpass filtered (0.5–20 MHz), and three wPAT images (20 × 20 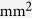 each) were reconstructed with Delay&Sum algorithm^35^,^36^ using data collected from the three ultrasound detection layers, respectively.

The oxygen saturation maps were calculated pixel by pixel via equation (1), and the pixels were chosen according to the calculated total hemoglobin (HbT) concentration (*C*_HbT_ = *C*_HbO_ + *C*_HbR_) which should be greater than 0.


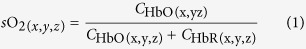



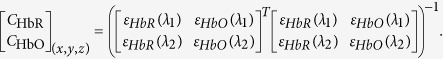



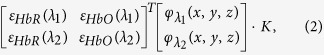


where, *C*_HbO_ and *C*_HbR_ are the concentration of HbO and HbR, *ε*_*HbO*_ and *ε*_*HbR*_ are the molar extinction coefficients (*cm*^−1^/M), 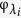 is the PA signal amplitude acquired at wavelength 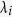, and *K* is the proportionality coefficient which is determined by acoustic parameters and the wavelength-dependent local light fluence^37^. It is worth noting that although due to the unknown coefficient *K* the calculated *C*_HbO_ and *C*_HbR_ are relative concentrations, the s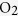 calculated from Equation 1 is absolute^38^.

For the visual stimulation experiments, the left primary visual cortex (LV1), right primary visual cortex (V2), and superior sagittal sinus (SSS) were chosen as regions of interest (ROI) 39. Photoacoustic (PA) signal amplitude across pixels in each ROI was averaged to obtain the cerebral hemodynamic response. The cerebral hemodynamic response time courses were calibrated according to the monitored light energy, and after subtracting and dividing the mean value of baseline (0–45 s, 100 images) they were converted into percentage time courses. Hemodynamic response time course (Fig. 5(d) and Sup Fig. 1(b)) for each wavelength was calculated by averaging across all 9 trials. A moving window averaging method (window length 5 s) was used to acquire the corresponding trend of time courses. Standard error of the mean (s.e.m.) was calculated for each run (n = 9) and superimposed to the averaged time course.

## Additional Information

**How to cite this article**: Tang, J. *et al.* Wearable 3-D Photoacoustic Tomography for Functional Brain Imaging in Behaving Rats. *Sci. Rep.* **6**, 25470; doi: 10.1038/srep25470 (2016).

## Supplementary Material

Supplementary Figure

## References

[b1] Kerr J. N. & Nimmerjahn A. Functional imaging in freely moving animals. Curr. Opin. Neurobiol. 22, 45–53 (2012). 22237048 10.1016/j.conb.2011.12.002

[b2] Schwarz C. *et al.* The head-fixed behaving rat—procedures and pitfalls. Somatosens Mot Res 27, 131–148 (2010). 20954892 10.3109/08990220.2010.513111 PMC3018133

[b3] Harvey C. D., Collman F., Dombeck D. A. & Tank D. W. Intracellular dynamics of hippocampal place cells during virtual navigation. Nature 461, 941–946 (2009). 19829374 10.1038/nature08499 PMC2771429

[b4] Szuts T. A. *et al.* A wireless multi-channel neural amplifier for freely moving animals. Nat. Neurosci. 14, 263–269 (2011). 21240274 10.1038/nn.2730

[b5] Miller E. K. & Wilson M. A. All my circuits: using multiple electrodes to understand functioning neural networks. Neuron 60, 483–488 (2008). 18995823 10.1016/j.neuron.2008.10.033

[b6] Lahti K. M., Ferris C. F., Li F., Sotak C. H. & King J. A. Imaging brain activity in conscious animals using functional MRI. J. Neurosci. Meth. 82, 75–83 (1998). 10.1016/s0165-0270(98)00037-5 10223517

[b7] Schulz D. *et al.* Simultaneous assessment of rodent behavior and neurochemistry using a miniature positron emission tomograph. Nat Methods 8, 347–352 (2011). 21399637 10.1038/nmeth.1582

[b8] Zhang T., Zhou J., Carney P. R. & Jiang H. Towards real-time detection of seizures in awake rats with GPU-accelerated diffuse optical tomography. J. Neurosci. Meth. 240, 28–36 (2015). 10.1016/j.jneumeth.2014.10.018 PMC4284948 25445250

[b9] Helmchen F., Fee M. S., Tank D. W. & Denk W. A miniature head-mounted two-photon microscope: high-resolution brain imaging in freely moving animals. Neuron 31, 903–912 (2001). 11580892 10.1016/s0896-6273(01)00421-4

[b10] Flusberg B. A. *et al.* High-speed, miniaturized fluorescence microscopy in freely moving mice. Nat. Methods 5, 935–8 (2008). 18836457 10.1038/nmeth.1256 PMC2828344

[b11] Sawinski J. *et al.* Visually evoked activity in cortical cells imaged in freely moving animals. Proc Natl Acad Sci USA 106, 19557–19562 (2009). 19889973 10.1073/pnas.0903680106 PMC2773198

[b12] Beard P. Biomedical photoacoustic imaging. Interface focus 1, 602–631 (2011). 22866233 10.1098/rsfs.2011.0028 PMC3262268

[b13] Kim J. Y., Lee C., Park K., Lim G. & Kim C. Fast optical-resolution photoacoustic microscopy using a 2-axis water-proofing MEMS scanner. Sci Rep 5, 10.1038/srep07932 (2015). PMC4300456 25604654

[b14] Xia J., Yao J. & Wang L. V. Photoacoustic tomography: principles and advances. *Electromagnetic waves* (Cambridge, Mass) 147, 10.1002/cmmi.443 (2014). PMC4311576 25642127

[b15] Prahl S. Optical absorption of hemoglobin. (2001) Available at: http://omlc.org/spectra/hemoglobin/ (Accessed: 16th December 2015).

[b16] Yao L., Xi L. & Jiang H. Photoacoustic computed microscopy. Sci Rep 4, 10.1038/srep04960 (2014). PMC4021812 24828539

[b17] Gamelin J. *et al.* A real-time photoacoustic tomography system for small animals. Opt Express 17, 10489–10498 (2009). 19550444 10.1364/oe.17.010489 PMC2757438

[b18] Xiang L. *et al.* Noninvasive real time tomographic imaging of epileptic foci and networks. Neuroimage 66, 240–248 (2013). 23128072 10.1016/j.neuroimage.2012.10.077

[b19] Tang J., Dai X. & Jiang H. Wearable scanning photoacoustic brain imaging in behaving rats. J. of Biophotonics 1, 10.1002/jbio.201500311 (2016). 26777064

[b20] Xiang L., Wang B., Ji L. & Jiang H. 4-D photoacoustic tomography. Sci Rep 3, 10.1038/srep01113 (2013). PMC3552346 23346370

[b21] Wang B. *et al.* Photoacoustic tomography system for noninvasive real-time three-dimensional imaging of epilepsy. Biomed Opt Express 3, 1427–1432 (2012). 22741087 10.1364/BOE.3.001427 PMC3370981

[b22] Öz G. *et al.* Neuroglial metabolism in the awake rat brain: CO2 fixation increases with brain activity. J. Neurosci. 24, 11273–11279 (2004). 15601933 10.1523/JNEUROSCI.3564-04.2004 PMC6730363

[b23] Masamoto K. & Kanno I. Anesthesia and the quantitative evaluation of neurovascular coupling. J Cereb Blood Flow Metab 32, 1233–1247 (2012). 22510601 10.1038/jcbfm.2012.50 PMC3390804

[b24] Austin V. *et al.* Confounding effects of anesthesia on functional activation in rodent brain: a study of halothane and α-chloralose anesthesia. Neuroimage 24, 92–100 (2005). 15588600 10.1016/j.neuroimage.2004.08.011

[b25] Tang J. *et al.* Noninvasive high-speed photoacoustic tomography of cerebral hemodynamics in awake-moving rats. J Cereb Blood Flow Metab, 35(8), 1224–32, 10.1038/jcbfm. (2015). 26082016 PMC4527999

[b26] Tian P. *et al.* Cortical depth-specific microvascular dilation underlies laminar differences in blood oxygenation level-dependent functional MRI signal. Proc Natl Acad Sci USA 107, 15246–15251 (2010). 20696904 10.1073/pnas.1006735107 PMC2930564

[b27] Peirce J. W. PsychoPy—psychophysics software in Python. J Neurosci Meth 162, 8–13 (2007). 10.1016/j.jneumeth.2006.11.017 PMC2018741 17254636

[b28] Van Camp N., Verhoye M., De Zeeuw C. I. & Van der Linden A. Light stimulus frequency dependence of activity in the rat visual system as studied with high-resolution BOLD fMRI. J Neurophysiol 95, 3164–3170 (2006). 16394078 10.1152/jn.00400.2005

[b29] Schulze-Clewing J., Eberle M. J. & Stephens D. N. Miniaturized circular array [for intravascular ultrasound]. In: Ultrasonics Symposium, 2000 IEEE, 10.1109/ULTSYM.2000.921550 (2000).

[b30] Purinton D. L. Microelectronic assemblies including Z-axis conductive films. United States Patent US 5,818,700. 1998 Oct 6.

[b31] Foster F. S., Harasiewicz K. A. & Sherar M. D. A history of medical and biological imaging with polyvinylidene fluoride (PVDF) transducers. Ultrasonics, Ferroelectrics, and Frequency Control, IEEE Transactions on 47, 1363–1371 (2000). 10.1109/58.883525 18238682

[b32] Tang J. & Jiang H. Single Element-Based Dual Focused Photoacoustic Microscopy. Photonics 2, 156–163 (2015).

[b33] Azhari H. Basics of biomedical ultrasound for engineers. John Wiley & Sons (2010).

[b34] Standard A. Z136. 1–2000, for Safe Use of Lasers. In: *Published by the Laser* (2000).

[b35] Johnson D. H. & Dudgeon D. E. Array signal processing: concepts and techniques. Simon & Schuster (1992).

[b36] Kruger R. A., Reinecke D. R. & Kruger G. A. Thermoacoustic computed tomography–technical considerations. Med Phys 26, 1832–1837 (1999). 10505871 10.1118/1.598688

[b37] Maslov K., Sivaramakrishnan M., Zhang H. F., Stoica G. & Wang L. V. Technical considerations in quantitative blood oxygenation measurement using photoacoustic microscopy *in vivo*. In: *Biomedical Optics 2006* International Society for Optics and Photonics, 10.1117/12.646265 (2006).

[b38] Zhang H. F., Maslov K., Sivaramakrishnan M., Stoica G. & Wang L. V. Imaging of hemoglobin oxygen saturation variations in single vessels *in vivo* using photoacoustic microscopy. Appl. Phys. Lett. 90, 053901-053901–053903 (2007).

[b39] Pellegrino L. J. & Cushman A. J. A stereotaxic atlas of the rat brain. Appleton-Century-Crofts (New York, 1967).

