# Supplementary material for: Wearable 3-D Photoacoustic Tomography for Functional Brain Imaging in Behaving Rats
Source: Sci Rep. 2016 May 5;6:25470. doi: 10.1038/srep25470 (PMC4857106; doi:10.1038/srep25470)
Supplement: Supplementary Figure [file srep25470-s1.pdf]

Wearable 3-D Photoacoustic Tomography for Functional Brain Imaging in Behaving Rats

Jianbo Tang[1], Jason E. Coleman[2], Xianjin Dai[1], and Huabei Jiang[1,3]*

[1]J. Crayton Pruitt Family Department of Biomedical Engineering, University of Florida, Gainesville, FL 32611, USA
[2]Child Health Research Institute, Department of Pediatrics, College of Medicine , University of Florida, Gainesville, FL 32610, USA
[3]School of Physical Electronics, University of Electronic Science and Technology of China, Chengdu, Sichuan, China
*Corresponding Author: Huabei Jiang, E-mail: hjiang@bme.ufl.edu.

Supplementary figure

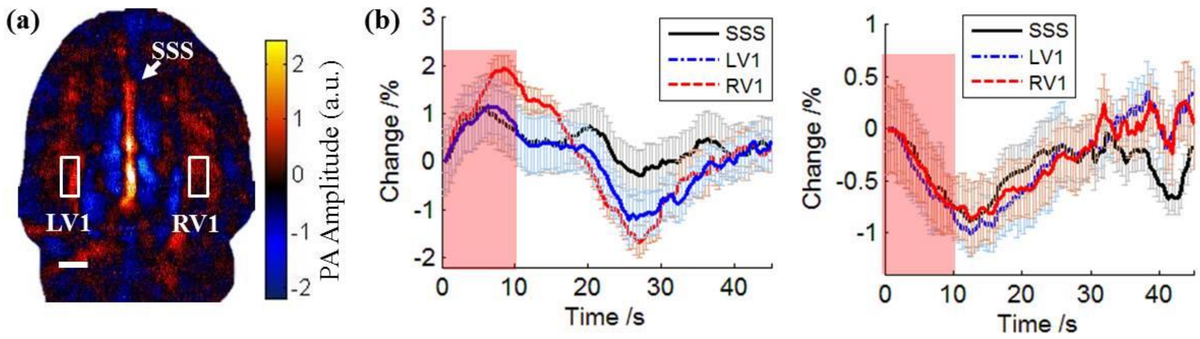

SupFig. 1 Visual stimulation result for rat #2

(a) The wPAT image from top layer (cortex layer). Three ROIs were marked on the wPAT image. RV1: right primary visual cortex (V1); LV1: left primary visual cortex (V1); SSS: superior sagittal sinus. (b) CBV response averaged across the 9 trials (superimposed with standard error of the mean, n=9) for all ROIs. Right, CBV response under 710 nm light excitation (HbR); Left, CBV response under 840 nm light excitation (HbO). Length of scalebar: 1 mm. The red shading represents 10 s stimulus.
